# Early progression of a pancreatic metastasis of synovial sarcoma after pancreatectomy

**DOI:** 10.1186/s40792-023-01612-z

**Published:** 2023-02-27

**Authors:** Mitsuyoshi Okazaki, Kaoru Katano, Hiroaki Sugita, Tomokazu Tokoro, Ryousuke Gabata, Satoshi Takada, Shinichi Nakanuma, Isamu Makino, Shintaro Yagi

**Affiliations:** Department of Hepato-Biliary-Pancreatic Surgery and Transplantation, Kanazawa University, 13-1 Takara-Machi, Kanazawa, Ishikawa 920-8641 Japan

**Keywords:** Synovial sarcoma, Pancreatic metastasis, Pancreatectomy

## Abstract

**Background:**

Synovial sarcoma is a malignant tumor that constitutes up to 10% of all soft-tissue sarcomas. The most frequent metastatic sites of synovial sarcoma are the lungs, lymph nodes, and bone, whereas pancreatic metastasis is extremely rare. Here, we report a case of pancreatic metastasis of synovial sarcoma.

**Case presentation:**

Nine years before presentation, a 31-year-old woman underwent extensive resection of the primary tumor after chemotherapy for left upper extremity synovial sarcoma. Six months before presentation, interscapulothoracic amputation was performed for an enlarged mass in the left upper extremity; the patient was treated with pazopanib. Three months before presentation, chest computed tomography showed multiple lung metastases; during subsequent follow-up, abdominal computed tomography revealed a pancreatic metastasis of synovial sarcoma. The doubling time of the pancreatic tumor was 14 days, and it grew rapidly. Furthermore, treatment-resistant pancreatitis symptoms were detected; thus, we performed distal pancreatectomy and administered one course of a 70% dose of trabectedin. However, the patient died of rapid progression of lung metastasis and respiratory failure within 2 months after surgery.

**Conclusions:**

Pancreatectomy may be carefully performed in cases of isolated pancreatic metastasis. However, the presence of other distant extrapancreatic metastases (e.g., uncontrolled lung metastases) may rule out pancreatectomy treatment.

## Background

Synovial sarcoma (SS) is a malignant soft tissue tumor that constitutes approximately 1% of all adult cancers and up to 10% of soft-tissue sarcomas [1–3]. The incidence of SS peaks in the first 30 years of life; these tumors affect the extremities and arise adjacent to large joints [4]. Because of the aggressive potential of SS, metastasis occurs in approximately 50% of patients; the most frequent sites of metastasis are the lungs, lymph nodes, and bone [4]. However, pancreatic metastasis rarely occurs in SS. Here, we report the clinicopathological features of a case of pancreatic metastasis of SS in a 31-year-old woman.

## Case presentation

**Fig. 1:**
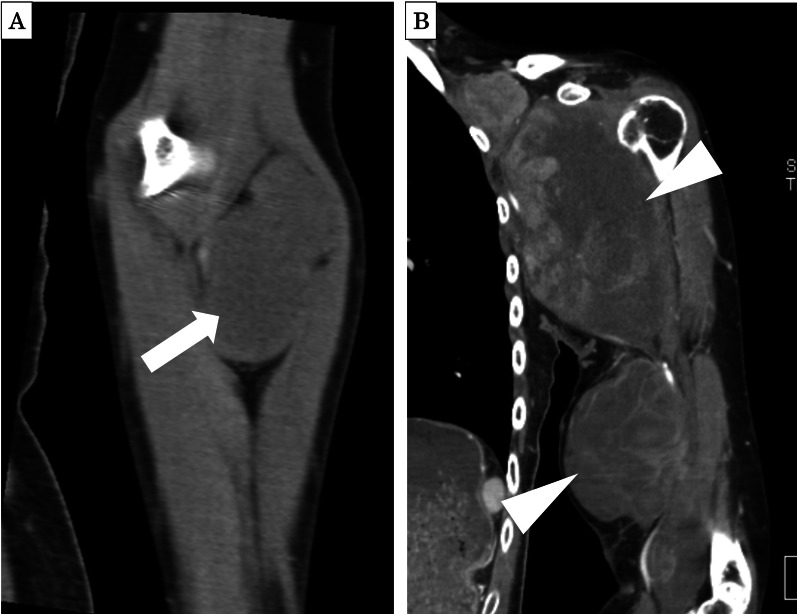
**A** Abdominal contrast-enhanced CT revealed a mass in the left upper extremity 9 years before presentation (arrow). **B** An enlarged mass was detected in the left upper extremity 6 months before presentation (arrowhead)

**Fig. 2:**
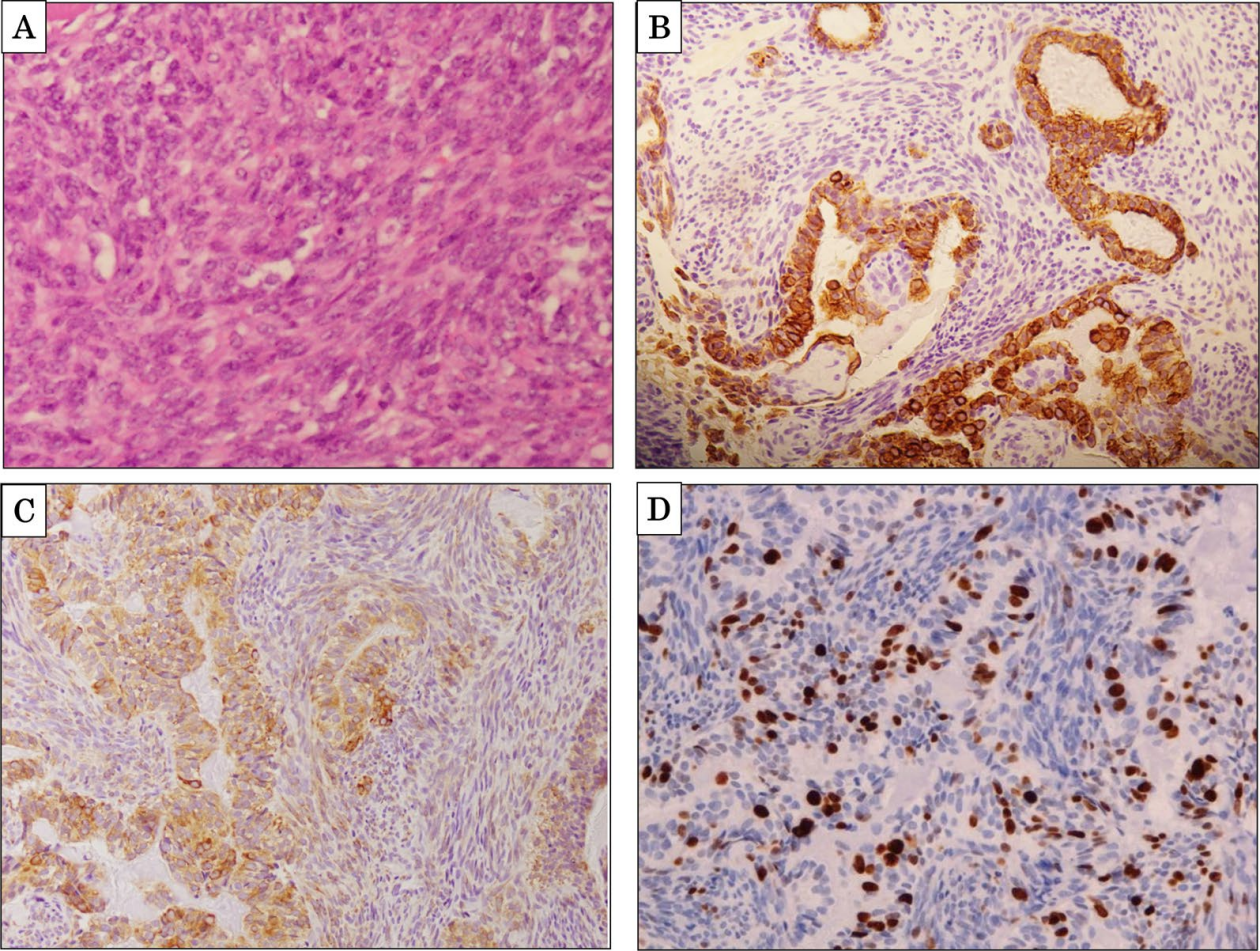
**A** Histological findings of the specimen revealed a biphasic-type tumor consisting of both epithelial- and spindle-shaped cells. Immunohistochemistry for cytokeratin (**B**), Bcl-2 (**C**), and MIB-1 (**D**)

**Fig. 3:**
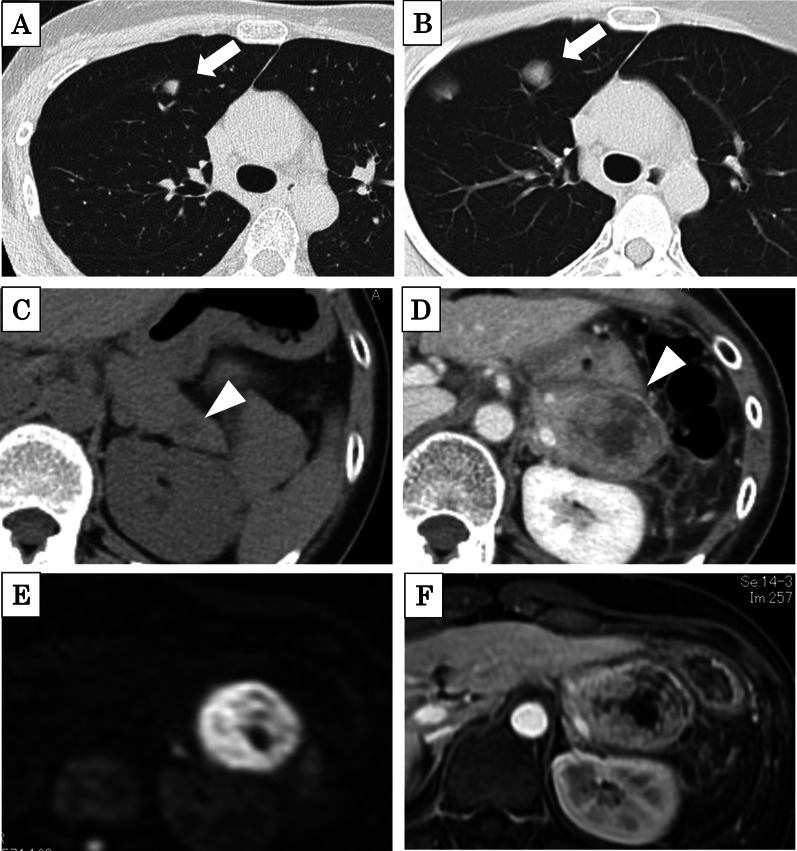
**A** Chest CT displayed multiple ≤ 18-mm lung metastases (arrow) 3 months before presentation. **B** Follow-up chest CT showed a 24-mm tumor (arrow) 1 month before presentation. **C** Retrospective review of abdominal non-contrast enhanced CT revealed a 9-mm tumor in the pancreatic tail (arrowhead) 3 months before presentation. **D** Abdominal contrast-enhanced CT revealed a 35-mm tumor at the time of presentation (arrowhead). **E** Abdominal MRI revealed a high-signal-intensity tumor on diffusion-weighted images, and **F** a contrast-enhanced tumor was detected on dynamic MRI

The patient was a 31-year-old woman who first visited our hospital 9 years ago prior to this case because of a heterogeneous low-density mass lesion without calcification had been detected in the left upper extremity on computed tomography (CT; Fig. 1A). She was diagnosed with left upper extremity SS. After chemotherapy, she underwent extensive resection of the primary lesion. Histological examination revealed a biphasic-type lesion consisting of both epithelial- and spindle-shaped cells on hematoxylin and eosin staining (Fig. 2A). Immunohistochemistry revealed positivity for cytokeratin AE1/AE3 and Bcl-2, and along with negativity for α-smooth muscle actin (SMA), desmin, S100, and CD34 (Fig. 2B, C). The MIB-1 proliferation index was approximately 10% (Fig. 2D). During follow-up, she underwent six rounds of chemotherapy and six mass resections for recurrent left axillary lymph nodes. Six months prior to this case, an enlarged mass was observed in the left upper extremity (Fig. 1B); the patient underwent interscapulothoracic amputation and was treated with pazopanib. Three months prior to this case, chest CT showed multiple ≤ 18-mm lung metastases (Fig. 3A); however, treatment was not modified at that time. One month prior to this case, follow-up chest CT showed a 24-mm tumor (Fig. 3B); the doubling time of the lung metastases was 30 days.

In this case, the patient presented with fever and left-sided abdominal pain; she was admitted to our institution for further investigation. Physical examination revealed tenderness and rebound tenderness on the left side of the abdomen.

Laboratory examination revealed elevated levels of C-reactive protein (2.4 mg/dL), amylase (315 IU/L), and lipase (1104 IU/L). The hemoglobin concentration; white blood cell and platelet counts; and electrolyte, liver enzyme, and bilirubin levels were within the reference ranges. The levels of tumor markers (e.g., carcinoembryonic antigen and carbohydrate antigen 19-9) were all within normal ranges.

Abdominal contrast-enhanced CT revealed a 37-mm tumor in the pancreatic tail, which exhibited heterogeneous low density with post-contrast enhancement and pancreatic parenchymal enlargement, as well as peripancreatic soft tissue inflammation. Retrospective review of abdominal non-contrast enhanced CT from 3 months prior revealed a 9-mm tumor (Fig. 3C, D). The doubling time of the pancreatic tumor was 14 days. Abdominal magnetic resonance imaging (MRI) revealed that the tumor in the pancreatic tail was similar to the tumor in the primary left upper extremity lesion, exhibiting a low signal on T1-weighted images, a slightly hyperintense signal on T2-weighted images, high signal intensity on diffusion-weighted images, and contrast enhancement on dynamic MRI (Fig. 3E, F).

The diagnosis was pancreatic metastasis of SS, with obstructive pancreatitis and progressive bilateral multiple lung metastases. The patient had multiple lung metastases; although additional drug therapy was planned, distal pancreatectomy was performed as palliative surgery because the tumor was growing rapidly and symptoms of treatment-resistant pancreatitis were present. Additionally, we were concerned that the rapidly enlarging tumor would rupture, leading to gastrointestinal perforation and peritoneal seeding, which would interfere with anticancer therapy.

**Fig. 4:**
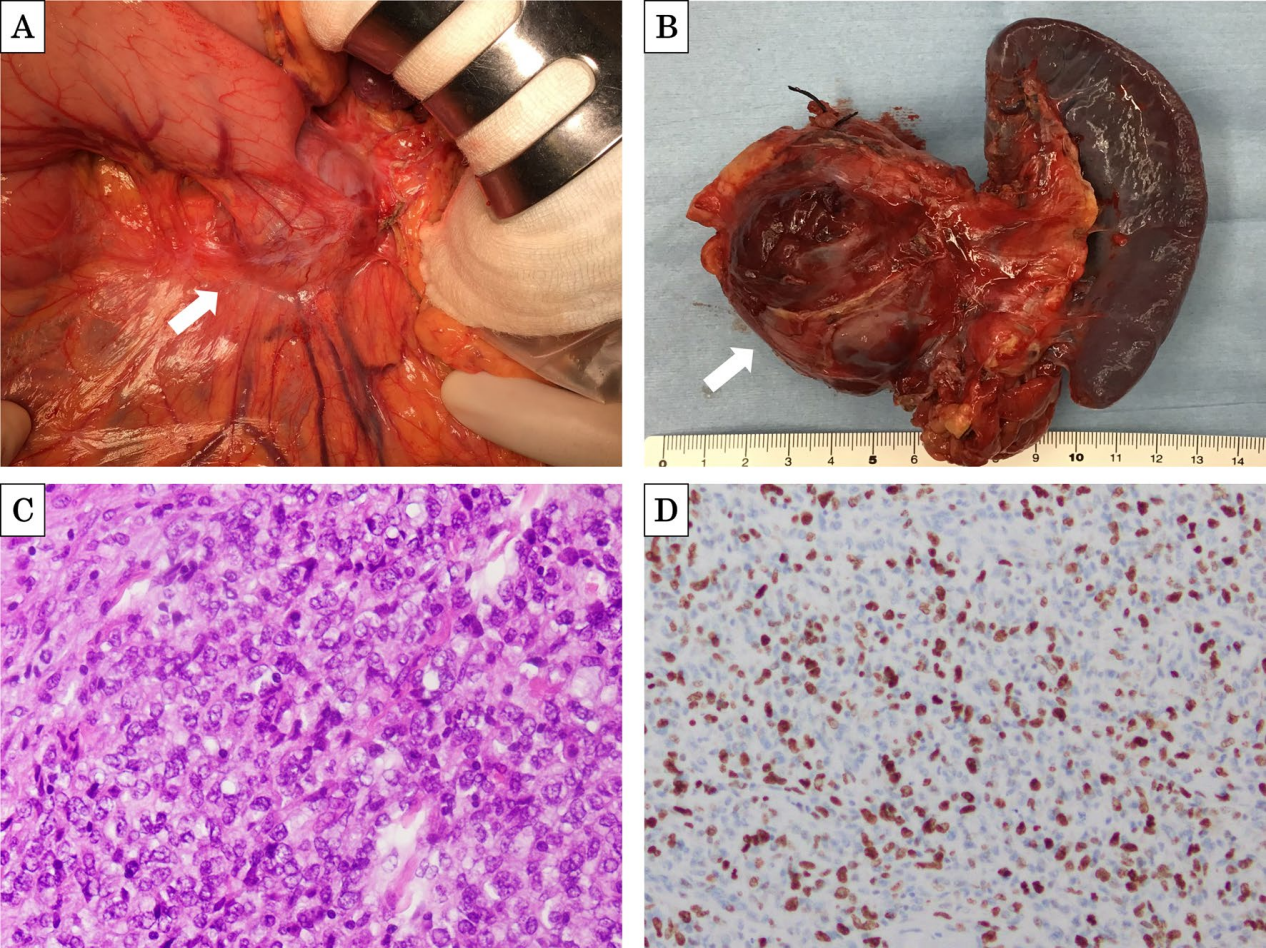
**A** Intraoperative view of the resected mass (arrow). **B** Surgical specimens of the pancreas revealed a well-circumscribed mass (arrow). **C** Histological findings of the specimen revealed a monophasic-type tumor. **D** Immunohistochemistry for MIB-1

After the omentum had been opened, intraoperative findings confirmed a tumor in the pancreatic tail without infiltration of the surrounding organs (Fig. 4A). The pancreas was dissected via linear stapling, and distal pancreatectomy was performed. Examination of the gross specimen revealed a well-circumscribed mass measuring 50 mm and occupying the pancreatic tail (Fig. 4B). Histopathological examination of the tumor uncovered the presence of a monophasic-type tumor consisting of both round and oval-shaped cells with a high nuclear/cytoplasmic ratio; there were no spindle-shaped cells on hematoxylin and eosin staining (Fig. 4C). The MIB-1 proliferation index was approximately 20% (Fig. 4D). Based on these findings, the patient was diagnosed with pancreatic metastasis of SS.

According to the International Study Group of Pancreatic Fistula classification [5], a grade B pancreatic fistula was observed, and it was conservatively treated. The patient was transferred to the Department of Orthopaedic Surgery for chemotherapeutic treatment of lung metastasis on postoperative day 28. The patient only received one course of a 70% dose of trabectedin because the lung metastasis rapidly progressed, and blood sputum was repeatedly observed. There was no intra-abdominal lesion, but the patient subsequently experienced rapid progression of the lung metastasis and died of respiratory failure within 2 months after surgery.

## Discussion

SS is a malignant neoplasm and the fourth most common soft-tissue sarcoma, which constitutes approximately 10% of all such lesions [1–3]. SS occurs mainly in the para-articular portion of tendons, tendon sheaths, and bursae structures in the lower extremities in 60–70% of cases [6]. Because of the aggressive potential of SS, metastasis occurs in approximately 50% of patients; the most frequent sites of metastasis are the lungs (74–81%), lymph nodes (3–23%), and bone (10–20%) [4]. SS tends to recur both early and late, with a 10-year disease-free survival (DFS) rate of approximately 50% [4].

Metastatic tumors in the pancreas are rare, and approximately 2% of pancreatic tumors are metastases from other primary sites [7, 8]. In a previous literature review of pancreatic metastases, most lesions were metastases of renal cell carcinoma (62.6–70.1%), followed by melanoma (4.0–9.1%), colorectal carcinoma (6.2–8.9%), and sarcoma (4.3–7.2%) [7, 8]. Pancreatic metastasis of SS is extremely rare; only seven cases have been reported worldwide, including our case, as summarized in Table 1 [9–14].

**Table 1:** Characteristics of previously reported cases and the present case of PM from synovial sarcoma

Authors	Year	Age, years	Sex	Age, years	Duration from primary to PM, years	Location	Histological subtype	Number of PMs	Size, mm	Location	Treatment	Extra pancreatic metastasis	Prognosis
Yamamoto et al. [9]	2001	40	Female	40	14	Right thigh	NA	1	UF	Head	PPPD	No	DFS = 6 years
Sandeep et al. [10]	2006	44	Female	44	10	Left thigh	Monophasic	1	80	Head	Biliary drainage	NA	NA
Somashekar et al. [11]	2014	38	Male	38	1	Right ilium, left glenoid	Monophasic	8	3–19	Tail	NA	Yes	NA
Makino et al. [12]	2016	36	Male	36	4	Left pelvis, femur	Monophasic	1	35	Body	LDP	No	DFS = 30 months
Bouhani et al.[13]	2020	30	Male	30	2	Left thigh	Monophasic	1	150	Tail	DP, colon resection	No	Died after 6 months after surgery
Yokose et al. [14]	2020	27	Female	27	4	Left femur	Monophasic	1	35	Tail	DP	No	RFS = 6 months
Our case	2022	31	Female	31	9	Left upper extremity	Monophasic	1	50	Tail	DP	Yes (lungs)	Died 2 months after surgery

*PM* pancreatic metastasis, *NA* not available, *PPPD* pylorus preserved pancreaticoduodenectomy, *LDP* laparoscopic distal pancreatectomy, *DP* distal pancreatectomy, *DFS* disease-free survival, *RFS* relapse-free survival

These cases comprised three men and four women; the median age at the diagnosis of pancreatic metastasis was 36 years (range, 27–44 years). The median time from resection of the primary tumor to the diagnosis of pancreatic metastasis was 4 years (range, 1–14 years). Pancreatic resection was performed in five patients, and three patients without distant metastasis had no recurrence (patient 1, DFS = 6 years; patient 2, DFS = 30 months; and patient 3, recurrence-free survival = 6 months). Excluding our case, no patients with pancreatic metastases of SS and extrapancreatic metastases underwent pancreas resection.

Soft-tissue sarcomas often have hematogenous distant metastases. However, the mechanism of metastasis to the pancreas is not fully understood. Bouhani et al. reported that the primary tumor was located in the lower limbs of all patients in their study; thus, they suspected that a shunt was present between the lower limbs and the pancreas [13]. However, the primary tumor in our case was located in an upper extremity. Further clarification of the pathogenesis is needed.

Because of the aggressive potential behavior of SS, radiological assessment is important for lesion staging and evaluation to identify the most appropriate therapy [15]. However, the features of imaging findings are not pathognomonic for pancreatic metastasis of SS. Past studies identified SS as a noninfiltrative, heterogeneously enhanced, well-circumscribed mass [2, 3]. Calcification is identified at the primary site in up to 30% of cases of SS [2]. The MRI features of the pancreatic metastasis include a heterogeneous mass, hypointensity on T1-weighted images, and hyperintensity on T2- and diffusion-weighted images [2, 3]. Positron emission tomography–CT may be helpful for prognosis because a pretreatment fluorodeoxyglucose avidity score of > 4.4 is reportedly associated with increased risks of local recurrence and metastasis of SS [15]. Furthermore, preoperative histological analysis is necessary for the diagnosis. We could not perform these additional examinations because of the need for semi-emergency surgery and the lack of time for examination related to the rapid increase in tumor size and the presence of pancreatitis symptoms.

SS is histologically divided into three subtypes: a monophasic type consisting only of spindle cells (50–60%), a biphasic type consisting of both epithelial and spindle cell components (20–30%), and a poorly differentiated type (15–20%) [2, 3]. On immunohistochemistry, SS is positive for epithelial markers such as keratins and epithelial membrane antigen, as well as Bcl-2; it is negative for skeletal muscle markers such as desmin, α-smooth muscle actin, and CD34 [16]. However, no distinctive markers for SS have been recognized [17]. In our case, histological assessment revealed that the primary tumor was the biphasic type; however, pancreatic metastasis features biphasic obscuration and changes in cell morphology. Furthermore, the MIB-1 proliferation index increased from approximately 10% to 20%. This finding indicated gradual changes in some prior resection specimens of local recurrence in the left upper extremity.

It is questionable whether pancreatic resection can result in long-term survival among patients with pancreatic metastases of SS. In cases of pulmonary metastasis of SS, long-term survival after pulmonary resection can only be expected if complete resection is achieved [18, 19]. Oliver et al. identified four factors that were associated with a good prognosis after surgical resection of pancreatic metastases of various malignant tumors: primary renal cell carcinoma, a > 3-year interval between resection of the primary tumor and the development of pancreatic metastases, isolated pancreatic metastases, and no prior recurrence [20]. Concerning SS, there is insufficient evidence to support treatment guidelines or determine the effectiveness of pancreatic resection for metastatic tumors because few patients undergo pancreatectomy to treat pancreatic metastases of SS. Our case met only one of the four Oliver criteria, and there was a need to control abdominal pain attributable to rapid tumor growth and pancreatitis symptoms. Furthermore, we were concerned that tumor rupture, gastrointestinal perforation, and peritoneal seeding would interfere with anticancer drug therapy. However, our patient subsequently experienced rapid progression of the lung metastasis and died of respiratory failure within 2 months after surgery. It may have been better to perform laparoscopic surgery to shorten the patient’s postoperative hospital stay. In a study of trabectedin outcomes among patients with unresectable and relapsed soft-tissue sarcomas, Kobayashi et al. reported that the median progression-free survival for the entire study cohort was 3.7 months, and the 6-month progression-free survival rate was 44% [21] In the present case, we regret that we performed pancreatectomy as palliative surgery; we speculate that drug therapy would have been better in this situation.

## Conclusion

We have presented a rare case of pancreatic metastasis of SS that was treated by distal pancreatectomy; however, the patient died of respiratory failure within 2 months after surgery because of rapid progression of the lung metastasis. Pancreatectomy may be carefully performed in cases of isolated pancreatic metastasis. However, the presence of other distant extrapancreatic metastases (e.g., uncontrolled lung metastases) may rule out pancreatectomy treatment.

## Data Availability

The datasets used and/or analyzed during the current study are available from the corresponding author on reasonable request.

## Abbreviations

SS: Synovial sarcoma
CT: Computed tomography
MRI: Magnetic resonance imaging
DFS: Disease-free survival
